# Initiation of breastfeeding within one hour of birth among mothers with infants younger than or equal to 6 months of age attending public health institutions in Addis Ababa, Ethiopia

**DOI:** 10.1186/s13006-018-0146-0

**Published:** 2018-01-23

**Authors:** Meseret Ekubay, Aster Berhe, Engida Yisma

**Affiliations:** 1 Department of Nursing, College of Health Sciences, Institute of Medicine and Health Sciences, Debre Berhan University, Debre Berhan, Ethiopia; 2 Midwifery Advisor, UNFPA Ethiopia Country Office, Addis Ababa, Ethiopia; 3 School of Allied Health Sciences, College of Health Sciences, Addis Ababa University, Addis Ababa, Ethiopia; 4 Robinson Research Institute, School of Medicine, The University of Adelaide, Adelaide, Australia

**Keywords:** Initiation of breastfeeding, Public health institution, Addis Ababa, Ethiopia

## Abstract

**Background:**

Breast milk is comprised of the essential nutrients that an infant needs in the first six months of life. Timely initiation of breastfeeding guarantees that infants receive the colostrum, ‘the first breastmilk’, which contains antibodies that protect the newborn against diseases. Breastfeeding within the first hour of life prevents newborn death due to sepsis, pneumonia, diarrhea and hypothermia. Although breastfeeding is a common practice in sub-Saharan Africa, evidence show that early initiation of breastfeeding is low.

**Methods:**

We conducted a cross-sectional study of 583 mothers with infants younger than or equal to 6 months of age attending Maternal and Child Health (MCH) clinics of public health institutions in Addis Ababa, Ethiopia from April to May 2012. A simple random sampling design was used to select the institutions included in this study. Data from mothers of infants were collected using interviewer-administered questionnaire. We analyzed the data to examine factors associated with initiation of breastfeeding within one hour of birth using logistic regression models.

**Results:**

Of 564 (96.7%, 564/583) mothers who breastfed their infants, 58.3% (329/564) initiated breastfeeding within one hour of birth. In the adjusted analysis, mothers who had three or more infants had about twice higher odds of timely initiation of breastfeeding within one hour of birth (Adjusted Odds Ratio [aOR] 2.10; 95% Confidence Interval [CI]1.04, 4.30) compared with mothers who had one infant. Furthermore, women who started antenatal care at their fourth month of pregnancy or later had a 49.0% higher odds of initiation of breastfeeding within one hour of birth (aOR 1.49; 95% CI 1.01, 2.19) compared to mothers who started antenatal care before their fourth month of pregnancy.

**Conclusions:**

Initiation of breastfeeding within one hour of birth was low. Initiation of breastfeeding within one hour of birth was highest among multiparous women, mothers aged 30–34 years, and women who began antenatal care at their fourth month of pregnancy or later. Public health officials and health care providers should consider interventions to promote and support early initiation of breastfeeding.

## Background

More than four million neonatal deaths occur every year, and most of these deaths are from low- and middle-income countries [1]. Breastfeeding within the first hour of life provides protection against infection and has been shown to prevent neonatal death due to sepsis, pneumonia, diarrhea and hypothermia [2–4]. Conversely, it was found that delayed initiation of breastfeeding after the first hour of birth doubled the risk of neonatal mortality [5].

The World Health Organization (WHO) recommends initiation of breastfeeding within one hour of birth [6]. However, the proportion of early initiation of breastfeeding among mothers varies widely across countries and regions worldwide. Factors that are often associated with timely initiation of breastfeeding include mother’s education [7–9], place of residence [10, 11], household income [12, 13], Place of birth [14], mother’s occupation [12, 15], cultural beliefs and/or traditional feeding practices [4, 16–18], counselling services provided during antenatal and postnatal visits [10, 13], and parity [9, 12], although these factors may differ according regions and states.

Previous studies revealed that cultural beliefs and traditional feeding practices may prevent mothers initiating breastfeeding immediately postbirth in developing settings. For example, in one systematic review involving 25 observational studies, it was found that women initiated breastfeeding in the evening after seeing stars if the child was born in morning due to cultural beliefs, and women discarded colostrum because of the negative perception that it may harm the newborn [4]. Furthermore, Legesse and colleagues in their cross-sectional study conducted in North-eastern Ethiopia demonstrated that late initiation of breastfeeding was associated with prelacteal feeding practice such as raw butter feeding [18], which is typically administered via a finger and perceived to be good for the health of newborns instead of colostrum.

In Addis Ababa, Ethiopia about 62.3% of births were born in public health institutions [19]. In 2004, the Family Health Department of Federal Ministry of Health of Ethiopia developed a national strategy for infant and young child feeding which states breastfeeding should begin within one hour after birth [20]. However, in Addis Ababa, there is limited insight regarding the prevalence and factors associated with timely initiation of breastfeeding among urban mothers. The purpose of this study was to estimate the prevalence and examine factors associated with initiation of breastfeeding within one hour of birth among urban mothers with infants younger than or equal to 6 months of age attending public health institutions in Addis Ababa, Ethiopia.

## Methods

### Study setting

This study was conducted in Addis Ababa, the capital city of Ethiopia and seat of African Union and United Nations World Economic Commission for Africa. The study participants were recruited at Maternal and Child Health (MCH) clinics of public health institutions in Addis Ababa.

### Study design

We used a cross-sectional study design and interviewer-administered questionnaires to collect data from mothers of infants younger than or equal to 6 months of age attending MCH clinics of public health institutions in Addis Ababa. Mothers who gave birth within the six months prior to the study were included in order to reduce the bias that may occur due to data collection with a longer recall period.

### Sample size and sampling

Sample size was estimated using a single population proportion formula and calculated with the following assumptions: 95% confidence level, 5% margin of error, and 62% expected prevalence of initiation of breastfeeding within one hour of birth among mothers in public health institutions in Addis Ababa, Ethiopia (based on a Demographic and Health Survey [DHS] finding reported in Ethiopia) [20]. Given these assumptions, the required sample size was found to be 362. Considering a conservative design effect of 1.5 for complex sampling, and a non-response rate of 10%, the final estimate was 597. Thus, a total of 597 mothers of infants attending MCH clinic of public health institutions in Addis Ababa were identified.

The total number of public health institutions in Addis Ababa was 49 (13 hospitals and 38 health centers). Of these, 10 health centers and 2 hospitals (i.e., a total of 12 public health institutions) were selected by simple random sampling design. The sample was then allocated to the institutions included in the current study proportional to the number of mothers attending MCH clinics of the respective health institutions. Average daily records of the number of clients available at MCH clinics were used in each institution for this purpose. And, at each study center, all mothers with infants younger than or equal to six months of age and presented to the MCH clinics of public health institutions from April to May 2012 were interviewed.

### Data collection

The questionnaire for this study was adapted from the Ethiopian 2011 DHS [20] and through review of relevant studies. The questionnaire was initially prepared in English and translated into Amharic. Before the actual data collection, the questionnaire was pretested for comprehension among mothers of infants younger than or equal to six months of age recruited from a nearby public health institution outside of the study area, and revisions were incorporated into the questionnaire. The data were collected by 10 health professionals (nurses and midwives) after two days training about the tool and techniques of data collection. Data collection sessions were arranged by communicating with concerned bodies in Addis Ababa health bureau and the selected public health institutions.

### Study variables

The outcome of interest was the proportion of mothers of infants who initiated breastfeeding within one hour of birth. Initiation of breastfeeding within one hour of birth was computed based on one question that enquires about the time at which mother’s recent infant was put to the breast immediately after birth for the first time. We examined how mother’s age, religion, ethnicity, marital status, media exposure, having home visit by health extension worker, parity, mother’s education, mother’s occupation, number of months pregnant at time of first antenatal care visit, mother’s attendance of health education/counselling, place of delivery, and household income affected the variability in initiation of breastfeeding within the first hour of birth.

In Ethiopia, health extension workers are salaried female community health workers with secondary-level school education who have completed a one-year training in basic health service delivery. They are selected from the community that they serve, to ensure local familiarity, and work at 75% time [21]. Mother’s attendance of health education/counselling was measured using 7 questions that entail whether or not health workers informed/counselled mothers regarding placing baby in skin-to-skin contact with the mother immediately after birth; timely initiation of breastfeeding; hand washing before taking care of baby; immunization; danger signs of the newborn; cord care; and care for low birth weight baby. Responses to these questions were dichotomized into satisfactory (when mothers answered correctly four or more questions) and unsatisfactory (less than four questions answered correctly). In order to rank monthly income status of study participants, we utilized the cutoff described by Mengesha et al. [22] as low (<500 ETB (Ethiopian birr)), medium (500–2000 ETB), and rich (>2000 ETB).

### Statistical analysis

The data were entered using Epi Info version 3.5.1 (CDC, Atlanta, GA, USA), and then exported to SPSS version 23.0 (IBM, Armonk, NY, USA) for further processing. All required variable recoding and transformation were done before the final data analysis. Frequency distributions, and cross-tabulations were used to describe the variables of the study. To examine factors associated with initiation of breastfeeding within one hour of birth, we calculated unadjusted and adjusted odds ratios (aOR) and their 95%CIs using logistic regression models. We identified variables that were associated with initiation of breastfeeding based on unadjusted analyses as well as through literature review**.** The variable incorporated in the final model based on unadjusted analyses include parity, number of months pregnant at time of first antenatal care visit, and mother’s attendance of health education/counselling. Mother’s age, mother’s education, and mother’s occupation were the other variables that were included based on the literature.

## Results

Of 597 mother–infant dyads approached, 583 mothers participated in the study (response rate 97.7%). The average age of participants was 25.7 years (± standard deviation =4.4 years). By ethnicity, the majority (47.86%) of the participants are Amhara and the majority of mothers (89.71%) were married. Initiation of breastfeeding within one of birth was highest among mothers in the age category of 30–34 years, mothers with secondary or more education, mothers who had two or more children, and women who started their first antenatal care visit at their fourth months of pregnancy or later. Compared with mothers who initiated breastfeeding after one hour of birth, more mothers who initiated breastfeeding within the first hour of birth gave birth at public hospitals and health centers. Details regarding characteristics of the study participants according to initiation of breastfeeding are given in Table 1.

**Table 1:** Characteristics of the study participants according to initiation of breastfeeding among mothers of infants attending public health institutions

Characteristics	Overall (*n* = 583)	Initiation of breastfeeding^a^	
		Within 1 h (*n* = 329)	After 1 h (*n* = 235)
Mother’s age			
15–19	33 (5.66)	17/32 (53.13)	15/32 (46.88)
20–24	202 (34.65)	117/196 (59.69)	79/196 (40.31)
25–29	222 (38.08)	117/217 (53.92)	100/217 (46.08)
30–34	116 (19.90)	72/110 (65.45)	38/110 (34.55)
35+	10 (1.72)	6/9 (66.67)	3/9 (33.33)
Religion			
Orthodox	398 (68.27)	232/383 (60.57)	151/383 (39.43)
Muslim	102 (17.50)	56/100 (56.00)	44/100 (44.00)
Protestant	80 (13.72)	40/78 (51.28)	38/78 (48.72)
Catholic	3 (0.51)	1/3 (33.33)	2/3 (66.67)
Ethnicity			
Amhara	279 (47.86)	168/269 (62.45)	101/269 (37.55)
Oromo	110 (18.87)	60/108 (55.56)	48/108 (44.44)
Tigre	49 (8.40)	25/47 (53.19)	22/47 (46.81)
Gurage	95 (16.30)	50/92 (54.35)	42/92 (45.65)
Others^b^	50 (8.58)	26/48 (54.17)	22/48 (45.83)
Marital status			
Not married	32 (5.49)	19/29 (65.52)	10/29 (34.48)
Married	523 (89.71)	295/508 (58.07)	213/508 (41.93)
Divorced	26 (4.46)	14/25 (56.00)	11/25 (44.00)
Widowed	2 (0.34)	1/2 (50.00)	1/2 (50.00)
Mother’s education			
No education	67 (11.49)	38/66 (57.58)	28/66 (42.42)
Primary	143 (24.53)	73/139 (52.52)	66/139 (47.48)
Secondary or more	373 (63.98)	218/359 (60.72)	141/359 (39.28)
Mother’s occupation			
Employed	222 (38.08)	124/213 (58.22)	89/213 (41.78)
Not employed	361 (61.92)	205/351 (58.40)	146/351 (41.60)
Household income			
Low	63 (10.81)	38/60 (66.33)	22/60 (36.67)
Middle	437 (74.96)	241/426 (56.57)	185/426 (43.43)
High	83 (14.24)	50/78 (64.10)	28/78 (35.90)
Media exposure			
Yes	449 (77.02)	250/433 (57.74)	183/433 (42.26)
No	134 (22.98)	79/131 (60.31)	52/131 (39.69)
Had home visit by health extension worker			
Yes	132 (22.64)	245/437 (56.06)	192/437 (43.94)
No	451 (77.36)	84/127 (66.14)	43/127 (33.86)
Parity			
1	371 (63.64)	199/359 (55.43)	160/359 (44.57)
2	141 (24.19)	83/138 (60.14)	55/138 (39.86)
3+	71 (12.18)	47/67 (70.15)	20/67 (29.85)
Number of months pregnant at time of first antenatal care visit (*n* = 565)			
< 4	224 (39.65)	113/214 (52.80)	101/214 (47.20)
4+	341 (60.35)	204/332 (61.45)	128/332 (38.55)
Place of delivery			
Home	21 (3.60)	10/19 (52.63)	9/19 (47.37)
Public hospital	232 (39.89)	124/228 (54.39)	104/228 (45.61)
Private hospital	58 (9.95)	27/24 (50.00)	27/24 (50.00)
Health center	269 (46.24)	166/261 (63.60)	95/261 (36.40)
Others^c^	3 (0.51)	2/2 (100.00)	0 (0.00)

^a^**(***n* **=** 19) mothers of infants did not practice breastfeeding

^b^Include; Wolyita, Kanbata, Silete, Gamebella and Somali ethnicities

^c^Non-governmental organization and private clinics

### Breastfeeding practices

Of 583 participants, 564 (96.7%, 564/583) practiced breastfeeding. Of these 564 mothers, 58.3% (329/564) initiated breastfeeding within the first hour of birth. Of all breastfed mothers, 33.0% of them practiced mixed feeding.

### Factors associated with initiation of breastfeeding within one hour of birth

Compared to mothers in the age category of 15–19 years, mothers aged 20–24 years had about three times higher odds of initiation of breastfeeding within one hour of birth (adjusted odds ratio (aOR 2.85; 95%CI 1.09, 7.47). Mothers who had three or more infants had about twice higher odds of initiation of breastfeeding within one hour of birth (aOR 2.10; 95%CI 1.04, 4.30) compared to mothers who had one infant.

Mothers who started antenatal care at their fourth month of pregnancy or later had a 49.0% higher odds of initiating breastfeeding within one hour of birth (aOR 1.49; 95%CI 1.01, 2.19) relative to mothers who started antenatal care before their fourth month of pregnancy. Likewise, initiation of breastfeeding within one hour of birth was associated with mother’s attendance of health education/counselling (aOR 2.27; 95%CI 1.54, 3.36). Table 2 summarizes the results on factors associated with initiation of breastfeeding within one hour of birth.

**Table 2:** Logistic regression analysis showing the odds ratio for initiation of breastfeeding within one hour of birth among mothers in relation to mother’s characteristics

Predictor	% initiation of breastfeeding within 1 h (*n* = 564)	Unadjusted OR (95% CI)	Adjusted OR^a^ (95% CI)
Mother’s age			
15–19	3.0	1	1
20–24	20.7	1.31 (0.61, 2.77)	2.85 (1.09, 7.47)
25–29	20.7	1.03 (0.49, 2.17)	1.93 (0.72, 5.13)
30–34	12.8	1.67 (0.75, 3.70)	2.50 (0.87, 7.17)
35+	1.1	1.76 (0.37, 8.31)	1.79 (0.30, 10.43)
Mother’s education			
No education	6.7	1	1
Primary	12.9	0.78 (0.43, 1.43)	0.90 (0.46, 1.78)
Secondary or more	38.7	1.09 (0.64, 1.88)	1.36 (0.72, 2.55)
Mother’s occupation			
Employed	22.0	1	1
Not employed	36.3	1.00 (0.74, 1.42)	1.04 (0.70, 1.54)
Parity			
1	35.3	1	1
2	14.7	1.21 (0.81, 1.81)	1.04 (0.66, 1.64)
3+	8.3	1.89 (1.08, 3.32)	2.1 (1.04, 4.30)
Number of months pregnant at time of first antenatal care visit			
< 4	20.0	1	1
4+	36.2	1.42 (1.01, 2.01)	1.49 (1.01, 2.19)
Mother’s attendance of health education/ counselling^b^			
Unsatisfactory	14.4	1	1
Satisfactory	40.2	2.21 (1.52, 3.20)	2.27 (1.54, 3.36)

^a^Mutually adjusted; *n =* total number of mothers who experience breastfeeding

^b^See the methods section for description of how this variable was computed

## Discussion

This study assessed the prevalence and factors associated with initiation of breastfeeding within one hour of birth among mothers of infants younger than or equal to 6 months age. We found that 58.3% of mothers of infants initiated breastfeeding within one hour of birth, suggesting poor practice of timely initiation of breastfeeding among mothers. This figure was, however, somewhat higher than the findings reported in previous studies completed in Arba Minch (57.0%) [23] and Goba (52.0%) [10] towns in Ethiopia. Nonetheless, the finding of the present study is far from the WHO recommendation that states all mothers should initiate breastfeeding within the first hour of birth [6]. As our study focused on urban mothers living in Addis Ababa (the capital of Ethiopia), the prevalence should have been much higher than the prevalence that reported in other parts of Ethiopia. It is quite clear that urban mothers have increased access to health care and health literacy compared with those who live in small towns or rural areas. Thus, interventions such as health education to urban mothers are required to promote initiation of breastfeeding within one hour of birth.

The finding of this study showed that mothers who had three or more infants have about twice higher odds of initiation of breastfeeding within one hour of birth relative to mothers who had one infant. This is consistent with previous studies conducted in Nigeria [12] and Saudi Arabia [24], indicating that multiparty is associated with timely initiation of breastfeeding. Similarly, a study completed in Amibara district, Northeastern Ethiopia showed that mothers who have 2–3 children were less likely to initiate timely breastfeeding compared to those who had four or more children [9]. These findings may suggest that if a mother had more childbirth experience, the next infant is more likely to be put to the breast within one hour life.

Mothers who started antenatal care at their fourth month of pregnancy or later had a 49.0% higher odds of initiating breastfeeding within one hour of birth relative to mothers who started antenatal care before their fourth month of pregnancy. This may imply that health education/advice provided during antenatal visits at later months of pregnancy appear to be important. Likewise, the present study revealed that initiation of breastfeeding within one hour of birth was associated with mother’s attendance of health education/counselling, which is consistent with a study conducted in Goba town in Ethiopia [10], demonstrating that postnatal care advice/counseling about breastfeeding was associated with early initiation of breastfeeding. These findings suggest that health education/counseling is an important intervention for promoting early initiation of breastfeeding.

The study has notable limitations. First, the study was conducted five years ago and the prevalence of initiation of breastfeeding with one hour of birth reported in the current study might have changed in recent years. However, we believe that the findings are still relevant as the study was designed to include only urban mothers of infants presented to public health facilities, and examined factors associated with initiation of breastfeeding within one hour of birth. Besides, unlike few previous studies conducted in different parts of Ethiopia, the current study included urban mothers with infants younger than or equal to six months age, leaving little room for the possibility of recall/information bias. Secondly, as this study is confined to mothers of infants who attended public health facilities in Addis Ababa, the findings may not be generalizable to mothers who presented to private health facilities as well as to those who did not visit any other health institutions in Addis Ababa. Thirdly, as we did not collect information regarding expressed breast milk feeding, babies may be fed expressed breast milk and reported that they were breastfed by mothers, which technically may not be breastfeeding. Finally, this study did not collect data on history of method of birth, and as caesarean section may be associated with lack of early initiation of breastfeeding [4], this would have relevant to collect.

## Conclusions

Initiation of breastfeeding within one hour of birth was low among mothers with infants younger than or equal to 6 months of age attending public health institutions in Addis Ababa. However, as the data for this study was collected several years ago, the current prevalence of timely initiation of breastfeeding might have changed. Early initiation of breastfeeding was highest among mothers aged 30–34 years and multiparous mothers. Public health officials and health care providers should consider interventions to promote and support timely initiation of breastfeeding.

## Abbreviations

DHS: Demographic Health Survey
ETB: Ethiopian Birr
MCH: Maternal and Child Health
WHO: World Health Organization
